# CEPP: Canadian Extracorporeal Life Support (ECLS) Protocol Project

**DOI:** 10.1016/j.cjco.2022.02.005

**Published:** 2022-02-15

**Authors:** Andrew Fagan, Brian Grunau, Andrew Caddell, James Gould, Erin Rayner-Hartley, Yoan Lamarche, Gurmeet Singh, Dave Nagpal, Marat Slessarev

**Affiliations:** a Division of Critical Care Medicine, Western University, London, Ontario, Canada; b Department of Emergency Medicine and the Centre for Health Evaluation & Outcome Sciences, University of British Columbia, Vancouver, British Columbia, Canada; c Department of Emergency Medicine, Dalhousie University, Halifax, Nova Scotia, Canada; d Division of Cardiology, University of British Columbia, Vancouver, British Columbia, Canada; e Department of Cardiac Surgery, Montreal Heart Institute, Université de Montréal, Montreal, Quebec, Canada; f Department of Critical Care Medicine & Surgery, Division of Cardiac Surgery, Mazankowski Alberta Heart Institute, University of Alberta, Edmonton, Alberta, Canada; g Division of Cardiac Surgery, Western University, London, Ontario, Canada

## Abstract

**Background:**

Extracorporeal life support (ECLS) is associated with high morbidity and mortality. Complications and mortality are higher at lower-volume centres. Most Canadian ECLS institutions are low-volume centres. Protocols offer one way to share best practices among institutions to improve outcomes. Whether Canadian centres have ECLS protocols, and whether these protocols are comprehensive and homogenous across centres, is unknown.

**Methods:**

Purposeful sampling with mixed methods was used. A Delphi panel defined key elements relevant to the ECLS process. Documentation used in the delivery of ECLS services was requested from programs. Institutional protocols were assessed using deductive coding to determine the presence of key elements.

**Results:**

A total of 37 key elements spanning 5 domains (referral, initiation, maintenance, termination, and administration) were identified. Documentation from 13 institutions across 10 provinces was obtained. Institutions with heart or lung transplantation programs had more-complete documentation than did non-transplantation programs. Only 5 key elements were present in at least 50% of protocols (anticoagulation strategy, ventilation strategy, defined referral process, selection criteria, weaning process), and variation was seen in how institutions approached each of these elements.

**Conclusions:**

The completeness of ECLS protocols varies across Canada. Programs describe variable approaches to key elements. This variability might represent a lack of evidence or consensus in these areas and creates the opportunity for collaboration among institutions to share protocols and best practice. The key-element framework provides a common language that programs can use to develop ECLS programs, initiate quality-improvement projects, and identify research agendas.

## Résumé

**Introduction:**

L’assistance cardiorespiratoire extracorporelle (ACRE) est associée à des taux élevés de morbidité et de mortalité. Les taux de complications et de mortalité sont plus élevés dans les centres à volume plus faible. La plupart des établissements qui offrent l’ACRE au Canada sont des centres à volume faible. Les protocoles constituent un moyen de partager des pratiques exemplaires entre les établissements afin d’améliorer les résultats. On ignore si les centres du Canada ont des protocoles d’ACRE, et si ces protocoles sont exhaustifs et homogènes dans tous les centres.

**Méthodes:**

Nous avons utilisé un échantillonnage dirigé par méthodes mixtes. Le panel Delphi a défini les éléments fondamentaux pertinents au processus d’ACRE. La documentation utilisée pour la prestation de services d’ACRE a été demandée aux programmes. Nous avons évalué les protocoles des établissements au moyen du processus inductif de codification pour déterminer la présence d’éléments fondamentaux.

**Résultats:**

Nous avons relevé un total de 37 éléments fondamentaux couvrant cinq domaines (aiguillage, amorce, maintien, cessation et administration). La documentation provenait de 13 établissements de 10 provinces. Les établissements qui ont des programmes de transplantation cardiaque ou pulmonaire avaient une documentation plus complète que les programmes sans transplantation. Seuls cinq éléments fondamentaux étaient présents dans au moins 50 % des protocoles (stratégie d’anticoagulation, stratégie de ventilation, processus défini d’aiguillage, critères de sélection, processus de sevrage), et une variation était observée dans la façon dont les établissements considéraient chacun de ces éléments.

**Conclusions:**

Au Canada, l’exhaustivité des protocoles d’ACRE varie. Les programmes décrivent la variabilité des approches des éléments fondamentaux. Cette variabilité qui pourrait représenter le manque de données probantes ou de consensus dans ces domaines ouvre la voie à la collaboration des établissements au partage des protocoles et des pratiques exemplaires. Le cadre des éléments fondamentaux contribue à offrir un langage commun que peuvent utiliser les programmes pour élaborer des programmes d’ACRE, amorcer des projets d’amélioration de la qualité et établir des programmes de recherche.

The use of extracorporeal life support (ECLS) has increased in the past 2 decades. Extracorporeal membrane oxygenation (ECMO), a common form of ECLS, is associated with high morbidity and mortality,^1^ with variable survival to discharge of 60%, 43%, and 29% for veno-venous (VV)-ECMO, veno-arterial (VA)-ECMO, and extracorporeal cardiopulmonary resuscitation (eCPR) cases, respectively.^2^ In keeping with evidence from other areas of medicine, a low frequency of cases may contribute to poor outcomes.^3^ In Canada, more than half of ECLS centres perform fewer than 10 VV-ECMO and 10 VA-ECMO cases annually.^4^ Similarly, in Germany, the majority of ECLS cases are done in lower-volume centres (< 50 VA ECMO cases/year) despite higher complication rates and mortality in these centres.^5^ Although limiting ECLS to centres of excellence will increase case numbers and concentrate expertise, the emergent nature of ECLS and the expansiveness of Canadian geography mean that ECLS will continue to be offered at smaller centres and will remain a high-stakes low-frequency event. Given this constraint, alternative solutions to improve ECLS outcomes are needed.

Standard ECLS protocols may help reduce variation in care between larger and smaller centres and have the potential to improve patient outcomes. In some instances, a hub-and-spoke relationship exists, whereby smaller institutions may feed cases to larger centres. Harmonization of ECLS protocols could positively impact patient care consistency. Additionally, standard protocols for ECLS referral and initiation shorten the time to initiation,^6^ which in turn is associated with improved survival.^7^

Although direct evidence between protocol use and improved ECLS outcomes is lacking, it seems intuitive that implementing standard protocols may beneficially influence outcomes for these high-stakes low-frequency events and warrants further investigation. Notably, in other complex industries, establishing emergency operating procedures is an important component of ensuring safety and quality improvement.^8^^,^^9^

A recent survey of Canadian centres offering ECLS showed that programs lacked protocols for important aspects of ECLS delivery, despite offering these services (eg, eCPR).^4^ For centres with protocols, only limited data are available to guide which elements should be included and their implementation.^10^, ^11^, ^12^ Better characterization of existing protocols and an understanding of their usage within Canadian programs could improve ECLS service delivery across the nation.

In this study, a Delphi process (further described in the Materials and Methods section) established key elements to include within ECLS protocols. The prevalence of these key elements was determined, and areas of consensus and uncertainty within existing ECLS program documentation from 13 Canadian centres were described. These results provide much-needed evidence regarding the current state of ECLS programs across Canadian centres, highlighting areas for future research and quality improvement.

## Materials and Methods

### Study design

The study was reviewed by the Western University Health Sciences Research Ethics Board, and the board determined that board oversight is not required. We employed mixed methods to characterize the current state of Canadian ECLS protocols. Utilizing a list of Canadian institutions deemed capable of delivering ECLS services from previous unpublished work, program leads were contacted via e-mail to request documentation related to ECLS delivery (eg, policy and procedures, order sets, protocols, etc). Document analysis required determination of important criteria to include within protocols (eg, anticoagulation protocol, ventilation protocol, etc). A Delphi panel was used to define these key elements. Documentation was analyzed for the presence of key elements, and areas of consensus and uncertainty were described.

### Delphi panel methods

Given the lack of consensus outlining ideal Canadian ECLS protocol content, a Delphi panel was created to identify key elements that should be included. The RAND/University of California, Los Angeles Delphi Method used in similar studies was adapted for this project.^13^^,^^14^ A 9-member panel with expertise in critical care, cardiac surgery, cardiology, emergency medicine, and internal medicine was assembled for this process. Panel members represented relevant disciplines, geographic distribution, and institution types, including lung transplantation, cardiac transplantation, and cardiac surgery programs. The process was anonymous and was delivered electronically over 3 rounds. Table 1 provides characteristics of the Delphi panel.

**Table 1:** Characteristics of Delphi panel

Years in practice	Number of members	Gender	Specialty training of panelist			
			Cardiac surgery	Cardiology	Critical care	Emergency medicine
< 5	4	3 male, 1 female	1	2	3	1
5–10	2	2 male	1	—	2	—
> 10	3	3 male	2	—	2	1

Round 1 asked panel members to identify key elements of the ECLS protocol/program manual. Panel members were provided with documentation explaining the purpose of the research project, examples of possible key elements and how they will be used in the study, and relevant literature.^10^, ^11^, ^12^ Panelists were asked to identify any and all relevant key elements.

Round 2 of the Delphi process consisted of a survey that asked panelists to rate the importance of key elements generated in round 1. Similar key elements were combined along with descriptions. A 9-point Likert scale was used, and a key element was considered important if it obtained a median score of 7 or more.^13^ Panelists were required to provide rationale for scores of 5 or lower.

Round 3 required panelists to approve the final list of key elements. Panelists were provided with a list of approved and rejected key elements with low score rationale. All members approved of the final list without the necessity of further rounds.

### Protocol sampling

Contact information for program leads was available for 24 institutions from previous work. Program leads were contacted via e-mail and provided with the rationale for the project. Any documentation related to the delivery or administration of ECLS services was requested. Program leads were recontacted at regular intervals until a representative sample was obtained. A geographically diverse sample, representing ECLS-capable institutions was targeted, incorporating programs with differences in volume and program-development robustness. Centres were categorized as heart-lung transplantation capable, heart transplantation capable, and cardiac surgery only capable. Programs were recontacted until representation from all provinces was obtained.

### Analysis

Deductive coding was used to analyze documents for the presence or absence of key elements. The key elements and their descriptions were used as a coding framework.^15^ A key element was coded as present if the element was directly mentioned (eg, anticoagulation protocol), or if indirect evidence that it existed was present (eg, some documents had approval stamps, suggesting a regular internal review process). Any text that did not meet the predefined coding framework was highlighted through inductive coding, and a memo journal was maintained throughout the process.

These data were used to determine the completeness of protocols by institution type. Additionally, key elements were grouped by their prevalence across protocols. Key elements that were present in more than 50% of institutional protocols underwent further analysis. The coded data from each key element were extracted from the original protocols and organized by key element code. These data then underwent narrative description.

To ensure reliability of coding, 2 authors independently reviewed the coded protocols for accuracy. Member checking was also employed to ensure validity.^16^ The results and analysis were shared with the Delphi panel prior to publication. Delphi panel members represented stakeholders familiar with ECLS programs, with intimate knowledge of their institutional protocols. This group also contained representation from the Canadian Cardiovascular Critical Care Society and the Canadian Critical Care Society.

## Results

The Delphi process occurred over 4 months and yielded 37 key elements identified as important components of an ECLS protocol (Table 2). These were broadly classified into 5 domains of the ECLS process (referral, initiation, maintenance, discontinuation, and administrative). The final list was approved by all 9 members of the panel.

**Table 2:** List of domain and key elements identified by Delphi panel

Key element followed by description	Prevalence in protocols, %
**Referral phase**	
Patient selection criteria	50
*Inclusion/Exclusion for respiratory failure, cardiac arrest, and cardiogenic shock*	
Shock team	25
*Who evaluates referral? Single person on call, medical director, panel of experts, ECMO team/specialist, or dedicated shock team?*	
Defined referral process	58
*Presence of algorithms to assist with rapid decision making, specific referral process for each indication, process for EMS referral or interhospital referral*	
Prehospital protocol	17
*EMS referral and resuscitation process, process for transition from ACLS to ECLS*	
**Initiation phase**	
Initiation process	33
*Defined location and process, specifically addressing cannula choice, heparin timing and route, cannulation site, etc.*	
Peripheral hospital initiation	8
*Process for mobile ECLS team, interhospital transport, etc.*	
Identified roles and responsibilities	25
*Who should be present, roles, training, ACLS vs ECLS team, specific cannulators?*	
Cannulation protocol	33
*Who should do it? Preference for specific sites, choice of cannula sizes, sedation and paralysis during cannulation*	
Anticoagulation	33
*Type and timing during cannulation*	
Checklists	25
*Equipment and actions to be performed including role assignments, and potential plan for after-action reviews*	
Consent process	17
*Is consent required? Should it be? How is it obtained?*	
Post-initiation procedures	33
*Defined process for post-initiation procedures such as cath lab activation, lower-extremity reperfusion, intensive care unit parameters, etc.*	
**Maintenance phase**	
ECMO circuit monitoring	50
*How does general system monitoring and maintenance occur (eg, clots, pressures, etc). Who is responsible for this?*	
Anticoagulation	75
*Choice of maintenance anticoagulation and monitoring parameters. Management of potential challenges (heparin resistance), monitoring for complications (clots, heparin-induced thrombocytopenia, bleeding) and periprocedural anticoagulation management.*	
Hemodynamic management	17
*Targets for hemodynamic support, choice of inotropes/vasopressors, and fluid management*	
Ventilator management	58
*Ventilator management specific for each indication (eg, respiratory vs cardiac failure). Initial management and overall guiding principles.*	
Temperature management	17
*Does cooling occur post-arrest; how does it occur?*	
Bleeding management	17
Is there a protocolized approach to address bleeding?	
Emergency protocols	42
*Do protocols exist for predictable emergencies (eg, accidental decannulation, thrombosis, etc.)?*	
Intrahospital transport protocol	17
*How are patients transported safely, who isresponsible?*	
Defined process for LV unloading	8
*Is there a defined process for when and how this occurs?*	
MCS/shock team	42
*Daily rounds, MRP, etc.*	
Mobilization strategy	25
*Is there a defined strategy for safe PT and mobilization of ECLS patients?*	
**Discontinuation phase**	
Weaning protocol	58
*How, where, and parameters that prompt weaning including associated changes in ventilator settings and/or hemodynamic support*	
Process for device transition	0
*Is there a defined process for device transition (eg, durable ventricular assist device, central ECMO, etc.)*	
Discontinuation of anticoagulation	0
*Heparin reversal, transition to DVT prophylaxis*	
Prognostication	33
*Expected duration, neuroprognostication, definition of futility*	
End-of-life planning	17
*Defined process for recognizing this, approaching it with family, etc.*	
Organ donation/procurement	17
*Process for declaration, approach to family, procurement, etc.*	
**Administration phase**	
Education	17
*Certification, maintenance of competency, including simulation of common challenges*	
Quality assurance	25
*Annual review, annual reports, comparison against established registries*	
Availability of clinical expertise	42
*Availability of appropriate clinical expertise (ie, surgical services, perfusion, PCI capacity, etc.)*	
Additional support services	17
*Ethics, pastoral care, etc.*	
ECLS committee/governance	33
*Formal administrative structure in place for monitoring and administration of ECLS program*	
Research program	8
*Data reporting, ELSO database, etc.*	
Partnership with prehospital organizations	8
*Appropriate partnership with relevant stakeholders*	
Appropriate protocols in place prior to program initiation	42
*Do relevant protocols exist prior to offering ECLS services (ie, inclusion criteria and referral process, etc.)*	

ACLS, advanced cardiac life support; DVT, deep vein thrombosis; ECLS, extracorporeal life support; ECMO, extracorporeal membrane oxygenation; ELSO, extracorporeal life support organization; EMS, emergency medical services; LV, left ventricular; MCS, mechanical circulatory support; MRP, most responsible physician; PCI, percutaneous coronary intervention; PT, physical therapy.

There are 39 institutions in Canada capable of delivering ECLS.^4^ Program documentation was obtained from 13 institutions (33%), representing all provinces, 2 of 4 lung/heart transplantation centres (50%), 5 of 11 heart transplantation centres (without lung transplantation; 45%), and 6 of 24 cardiac surgery-only centres (25%). The documents that were provided varied among centres and included practice guidelines, protocols, policies and procedures, and order sets. Two programs lacked any documentation but were in the process of developing protocols. Four programs provided documentation organized into “program manuals.”

**Figure 1:**
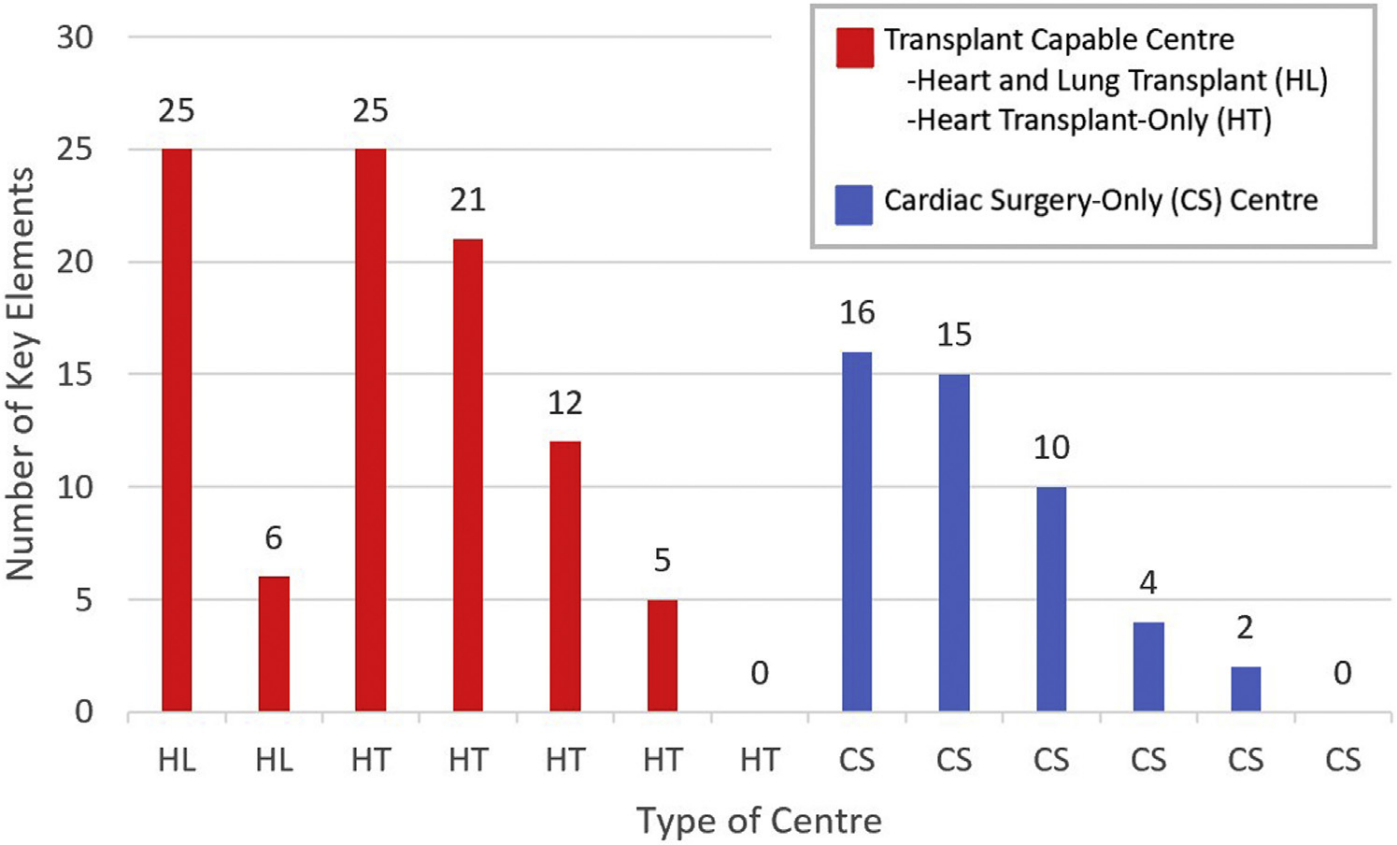
Number of key elements present in institutional protocols. Number of key elements by type of centre: HL; HT; and CS. **Red bars** represent transplantation-capable centres; **blue bars** represent CS centres.

Only 3 programs had documentation that included greater than 50% of the 37 key elements. Of these, one had a complete program manual in a single document that described roles and responsibilities, clinical management, relevant policies, and order sets. Two programs had a collection of clinical guidelines outlining the care process for specific ECLS phases (eg, initiation, weaning, etc). These documents provided clear roles and responsibilities of all team members, as well as the clinical rationale for appropriate management and troubleshooting during those phases. All centres that had more comprehensive documentation were either heart and/or lung transplantation capable centres (Fig. 1).

In regard to the programs with less-comprehensive documentation, variability was seen in the key elements that were addressed and in how they were addressed. Some programs had robust emergency ECLS procedures, whereas others had very detailed order sets with step-by-step nursing instructions. In contrast to comprehensive program manuals, these documents seemed more sporadic in the issues that were addressed. Documents more commonly focused on the roles of non-physician team members (eg, nursing, perfusion, and respiratory therapy).

### Descriptive review of common key elements

**Figure 2:**
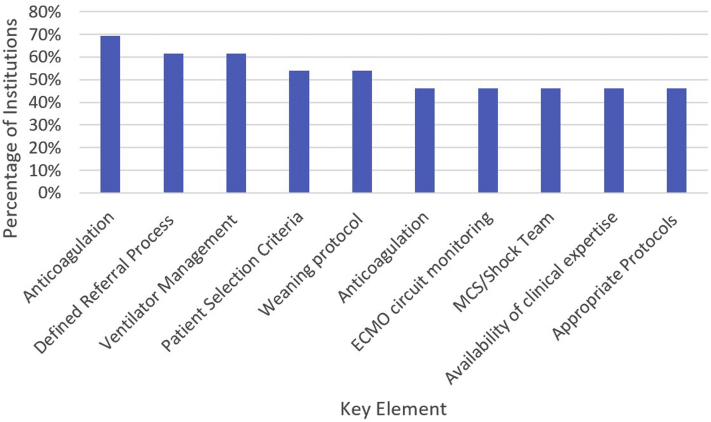
Prevalence of key elements found in protocols. ECMO, extracorporeal membrane oxygenation; MCS, mechanical circulatory support.

Figure 2 shows the prevalence of the 10 most common key elements. Five key elements were present in more than 50% of protocols (referral process, patient selection criteria, anticoagulation, ventilator management, and weaning protocol). Table 3, Table 4, Table 5, Table 6, Table 7, Table 8, Table 9 provide detailed description of how each key element was addressed by the institutions. Programs varied in how they address anticoagulation and ventilator management. Less variability was seen in how institutions addressed the key elements of patient selection, weaning protocol, and referral process.

**Table 3:** Key-element narrative review—defined referral process

Institution 1	Institution 2	Institution 3	Institution 5	Institution 6	Institution 7	Institution 12	Institution 13
For nonemergent cases, consensus between 2 consultant physicians (on-call surgeon) separate from MRP is required. Referral checklist that includes specific indications, contraindications, risk score calculations, and access-site availability.	Requires consensus of VAD team, CVICU, and cardiac surgery before proceeding with initiation of ECLS. Provides some guiding principles, including discussion around cardiogenic shock vs sepsis, eCPR and post–mitral valve surgery.	Requests cardiac surgery consultation for VA- and VV-ECMO. Mentions VAD team should also be consulted for VA-ECMO.	Process for emergency department initiation is identified. Emergency physician is to notify ICU consultant who will coordinate with required services (eg, perfusion, cardiac surgery, interventional cardiology).	Defined referral process specific to COVID19 pandemic. Flow chart outlining referral process, including inclusion/exclusion criteria and location-dependent activation pathway. ECMO triage team consists of relevant on-call personnel (eg, surgeon on call, ICU consultant, perfusion, etc.). This 6–7 member team must approve all ECMO activations and is responsible for overseeing active cases.	Well-defined referral process in flow chart form. Specific pathways for urgent vs emergent and cardiac vs respiratory failure. Provides inclusion/exclusion criteria, contact information, team members and process for transfer vs mobile ECLS.	eCPR activation form that includes inclusion vs exclusion criteria and specific process for code eCPR activation (eg, who to call, room setup, etc.)	Clearly defined referral process initiated through the on-call cardiac surgeon who provides initial screening before ECLS team is activated.

CVICU, intensive care unit; ECLS, extracorporeal life support; ECMO, extracorporeal membrane oxygenation; eCPR, extracorporeal cardiopulmonary resuscitation; ICU, intensive care unit; MRP, most responsible physician; VA, veno-arterial; VAD, ventricular assist device; VV, veno-venous.

**Table 4:** Key-element narrative review—patient selection for VA-ECMO

Institution 1	Institution 2	Institution 5	Institution 6	Institution 7	Institution 12	Institution 13
**Indications:** Cardiogenic shock with evidence of ongoing malperfusion eCPR and post-cardiotomy ECMO in highly selected patients. **Contraindications:** Not a transplant or VAD candidate (eg, cirrhosis, psychosocial issues) Sepsis is a relative contraindication SAVE score less than – 10 **Relative contraindications:** Nonrecoverable advanced comorbidity such as major CNS damage or terminal malignancy Contraindication to anticoagulation (eg, recent CNS hemorrhage or large ischemic stroke) Age > 75 y	**Indications:** Suggests referral for INTERMACS 1 and 2 or refractory cardiogenic shock Suggested indications include post-cardiotomy shock, acute MI, acute myocarditis, acute PE, circulatory support for PCI, preoperative support as a bridge to surgery, acute decompensation of chronic cardiomyopathy, severe accidental hypothermia **Contraindications:** None specified	**Indications:** Witnessed cardiac arrest, refractory to conventional ACLS Reversible etiology (eg, ACS, refractory dysrhythmia, PE, toxic ingestion, structural heart disease) Age < 65 y Refractory cardiogenic shock or recurrent arrests **Contraindications:** Delay in CPR > 5 min Duration of resuscitation > 45 min Significant pre-existing organ failure, active malignancy or DNR Active hemorrhage	**Indications:** None listed **Contraindications:** None listed	**Indications:** None listed **Contraindications:** Underlying condition with < 6 mo to live Immunocompromised Survival unlikely > 60 min CPR, severe lactic acidosis, unrecoverable multiorgan failure Severe vasodilatory shock Severe hemorrhage Coagulopathy Intracranial hemorrhage or uncontrolled extracranial hemorrhage Aortic dissection Not a VAD or transplant candidate (severe liver disease, ESRD, advanced malignancy) Recent advance directives Age > 70 y Substance abuse Known medical noncompliance Definite poor prognosis for neurologic recovery	**Indications (eCPR):** Arrest < 50 min Age < 65 y No major comorbidities (ESRD, liver failure, COPD, CHF) or pre-existing major neurologic deficits BMI appropriate for Lucas device No history or evidence of recreational drug use Nontraumatic arrest Witnessed arrest Initial shockable rhythm or signs of life with CPR Presumed cardiac arrest (ie, no obvious alternate cause) or overdose of cardiac toxins (eg, β-blocker) ETCO_2_ > 10 mm Hg	**Indications:** Post-cardiotomy: Failure to wean from bypass, low cardiac output, intractable dysrhythmia, pulmonary hypertension Nonsurgical cardiac failure: myocarditis, cardiomyopathy, low output syndrome Accidental hypothermia **Contraindication:** Advanced age > 65 y Weight < 40 kg BMI > 40 Femoral artery size < 5.5 mm Chronic organ dysfunction Prolonged CPR > 30 min Malignancy Clinically active bleeding Recent or expanding intracranial bleed Significant coagulopathy Immunosuppression (ANC < 400 mm^3^) Sepsis Severe irreversible brain damage Severe burn Clinical futility • Cardiac index < 1 L/min/m^2^ got VV-ECMO

ACS, acute coronary syndrome; ACLS, advanced cardiovascular life support; ANC, absolute neutrophil count; BMI, body mass index; CHF, congestive heart failure; CNS, central nervous system; COPD, chronic obstructive pulmonary disease; CPR, cardiopulmonary resuscitation; DNR, do not resuscitate; ECMO, extracorporeal membrane oxygenation; eCPR, extracorporeal CPR; ESRD, end-stage renal disease; ETCO_2_, end-tidal carbon dioxide; INTERMACS, Interagency Registry for Mechanically Assisted Circulatory Support; MI, myocardial infarction; PE, pulmonary embolism; PCI, percutaneous coronary intervention; SAVE, survival after VA-ECMO; VA, veno-arterial; VAD, ventricular assist device; VV, veno-venous.

**Table 5:** Key-element narrative review – patient selection for VV-ECMO

Institution 1	Institution 2	Institution 5	Institution 6	Institution 7	Institution 12	Institution 13
**Indications:** Refractory hypoxic or hypercarbic respiratory failure PaO_2_/FiO_2_ < 60 on FiO_2_ 100% and PEEP > 16; pH < 7.2, respiratory acidosis, regardless of PCO_2_ Acute or impending respiratory collapse (blocked airway, status asthmaticus that is unresponsive to optimal care) **Contraindications:** Mechanical ventilation on high settings for > 7 d; RESP score < – 6 **Relative contraindications:** Nonrecoverable advanced comorbidity such as major CNS damage or terminal malignancy Contraindication to anticoagulation (eg, recent CNS hemorrhage or large ischemic stroke) Age > 75 y	**Indications:** None listed **Contraindications:** None listed	**Indications:** None listed **Contraindications:** None listed	**Indications:** P/F ratio < 50 for 3 h P/F ratio < 80 for 6 h pH < 7.25 and pCO_2_ > 60 for 6 h with up to 35 breaths/min with plat pressure < 32 cm H_2_O **Contraindications:** Age > 65 y Duration mechanical ventilation > 7 d Pre-existing chronic lung disease requiring chronic O_2_ support or NIV support Moribund or SAPS-II > 90 Irreversible neurologic injury Coma following cardiopulmonary arrest Inadequate venous access Coagulopathy Severe distributive shock Severe coexisting comorbidities expected to limit life expectancy	**Indications:** None listed **Contraindications:** Signs of intracranial hemorrhage or other contraindication to anticoagulation HIT Lack of commitment to ongoing aggressive treatment Underlying condition limiting life expectancy Relative contraindications to consider (age > 55 y, BMI < 20, immunocompromised)	**Indications:** None listed **Contraindications:** None listed	**Indications:** < 7 d high setting mechanical ventilation Berlin definition of severe ARDS Reversible lung injury Isolated lung injury **Contraindication:** Advanced age > 65 y Weight < 40 kg BMI > 40 Femoral artery size < 5.5 mm Chronic organ dysfunction Prolonged CPR > 30 min Malignancy Clinically active bleeding Recent or expanding intracranial bleed Significant coagulopathy Immunosuppression (ANC < 400 mm^3^) Sepsis Severe irreversible brain damage Severe burn Clinical futility

ANC, absolute neutrophil count; ARDS, acute respiratory distress syndrome; BMI, body mass index; CNS, central nervous system; CPR, cardiopulmonary resuscitation; ECMO, extracorporeal membrane oxygenation; FiO_2_, fraction of inspired oxygen; HIT, heparin-induced thrombocytopenia; NIV, noninvasive ventilation; PaO_2_, partial pressure of oxygen; pCO_2_, partial pressure of carbon dioxide; PEEP, positive end-expiratory pressure; P/F, PaO_2_/FiO_2_; RESP score, respiratory ECMO survival prediction; SAPS, Simplified Acute Physiology Score; VV, veno-venous.

**Table 6:** Key-element narrative review—anticoagulation protocol

Institution 1	Institution 3	Institution 4	Institution 5	Institution 6	Institution 8	Institution 9	Institution 12	Institution 13
Heparin bolus 10,000–30,000 units given with cannulationDedicated heparin protocol with target PTT 50-64 s 5000–10,000 unit bolus is recommended for weaning flows below 1.5 LPM Bivalirudin or argatroban specified as alternate anticoagulation for HIT LMWH is suggested for stable patients	Reference to heparin protocol/order set is made, but no specific details for “non surgical” patients Implies case-by-case decision for post-cardiotomy anticoagulation	Refers to heparin protocol with specific monitoring parameters depending on the type of device PTT (range: 50–70.9 s) and Anti Xa (range: 0.3–0.5 U/mL) used for ECMO, Tandem Heart, Impella, Cardiowest, and Heartmate ACT (range: 160–180) used for ECMO, Tandem Heart, and Impella	5000-unit bolus of heparin given for cannulation No maintenance protocol, but refers to maintenance PPO that may contain this information	Recommends targeting ACT 160–200 s to be achieved with heparin	50–100 units/kg at the time of cannulation and continuous heparin infusion thereafter Recommends targeting ACT 1.5 × upper limit of normal for specific device (per ELSO) Then specifies targeting ACT 200–250 with ACT Plus or ACT 2; suggesting that typically this would be achieved with 20–40 units/kg/h	Starts at rate 15 units/kg/h with a target PTT of 50–70	Specifies heparin to be given on cannulation, but no further details Uses bivalirudin in cases of HIT	Daily checklist for anticoagulation referring to ACS protocol or provider-specified targets Prespecified parameters for INR and fibrinogen aimed at correcting coagulopathy

ACS, acute coronary syndrome; ACT, activated clotting time; ECMO, extracorporeal membrane oxygenation; ELSO, Extracorporeal Life Support Organization; HIT, heparin-induced thrombocytopenia; INR, international normalized ratio; LMWH, low-molecular-weight heparin; LPM, liters per minute; PPO, preferred provider organization; PTT, partial thromboplastin btime.

**Table 7:** Key-element narrative review—ventilator management

Institution1	Institution 3	Institution 4	Institution 6	Institution 12	Institution 13
Discusses philosophy of lung-protective ventilation, but acknowledges lack of evidence Provides initial strategy using PC ventilation targeting PC + PEEP < 30 cm H_2_O, PEEP 10–14, rate 4–8, Vt 3–4 mL/kg, FiO_2_ < 50%, O_2_sat > 85%, pH > 7.25 Can consider extubation in select cases with goal of liberating sedation, etc.	Suggests protective lung strategies should be employed with patients on VV-ECMO Specifically suggest PC mode with Pinsp < 25 cmH^2^, RR 8–10, PEEP 8–12, FiO_2_ < 0.3	Order set is provided for physician to prescribe ventilator settings	Provides suggested parameters aimed at preventing ventilator-induced lung injury Specifically suggests Pinsp < 20–25 cm H_2_O, PEEP < 10–15 cm H_2_O, FiO_2_ 0.3–0.4, O_2_sat > 85%, avoid recruitment maneuvers Goal PaO_2_ > 60 mm Hg, PaCO_2_ adjust sweep to achieve pH 7.35 –7.45	Suggests PC or VC aimed at lung-protective strategy Specifically suggests tidal volume 4–6 mL/kg with plateau pressures < 25 cm H_2_O, PEEP 5–10 cm H_2_O, FiO_2_ < 0.5 Consider recruitment maneuvers if indicated only after acute lung inflammation has subsided. Physician order is required.	Ventilator parameters chosen at the discretion of the ECLS team with no prespecified parameters

ECLS, extracorporeal life support; ECMO, extracorporeal membrane oxygenation; FiO_2_, fraction of inspired oxygen; O_2_ sat, oxygen saturation; PaCO_2_, partial pressure of carbon dioxide; PaO_2_, partial pressure of oxygen; PC, pressure-controlled; PEEP, positive end-expiratory pressure; Pinsp, inspiratory pressure; RR, respiratory rate; VC, vital capacity; VV, veno-venous.

**Table 8:** Key element narrative review—weaning protocol for VA-ECMO

Institution 1	Institution 2	Institution 3	Institution 4	Institution 6	Institution 12
Daily assessment by team for weaning appropriateness Gradual ECMO flow decrease to 2 LPM. Further weaning should be done in conjunction with echocardiographic assessment and with heparin bolus for flows < 1.5 LPM.	Gradually wean flows in 0.5 L increments to an idle flow of 2 LPM or 2.5 LPM if not adequately anticoagulated Can briefly decrease flow to 1.5 LPM to facilitate echocardiographic assessment if adequately anticoagulated. Once flows reduced, wean circuit FiO_2_ and sweep gas q2h to patient SvO_2_ 60–70, lactate < 2, and normal PA PO_2_ (100–190). Once cardiac function is improved and decannulation is planned, maintain circuit flow at minimum 2 LPM until decannulation Ventilator settings must be maintained below (fiO_2_ < 0.5, Pplat < 25, PEEP < 12)	Process described only for VV-ECMO	Provides hemodynamic and echo criteria to consider weaning. Hemodynamic: • Pulse pressure > 20 mm Hg for 24 h • MAP > 60 with no vasopressors or low dose of a single vasopressor. No inotropes. • CVP < 18–20 mm Hg Echocardiographic: •LVEF > 20-30% •LVOT VTI > 10 cm •PF > 200 Provides parameters for monitoring post decannulation.	Process described only for VV-ECMO	Suggest consider weaning when patient shows signs of recovery such as pulsatility or recovery on ECHO No specific details about assessment for weaning

CVP, central venous pressure; ECHO, echocardiogram; ECMO, extracorporeal membrane oxygenation; FiO_2_, fraction of inspired oxygen; LPM, liters per minute; LVEF, left ventricular ejection fraction; LVOT VTI, left ventricular outflow tract velocity time integral; MAP, mean arterial pressure; PA, pulmonary artery; PaO_2_, pulmonary artery partial pressure of oxygen; PEEP, positive end-expiratory pressure; PF, PaO_2_/FiO_2_; Pplat, plateau pressure; SvO_2_; venous oxygen saturation; VA, veno-arterial; VV, veno-venous.

**Table 9:** Key-element narrative review—weaning protocol for VV-ECMO

Institution 1	Institution 2	Institution 3	Institution 4	Institution 6	Institution 12
Daily assessment by team for weaning appropriateness Perfusionist weans oxygen flow and sweep, while respiratory therapist optimizes ventilator parameters	Daily assessment of need for ECMO Wean flows to 2 LPM or 2.5 LPM if not anticoagulated Wean FiO q2h for SaO_2_ > 92% ABG q4h to wean sweep for CO_2_ 35–45 Once FiO_2_ at 0.21 and sweep flow at 0.05-.1L, cap oxygenator Observe patient for at least 12 h (preferably 24) before consideration of decannulation Ventilator settings must be maintained below (fiO_2_ < 0.5, Pplat < 25, PEEP < 12)	Decrease pump flows incrementally by 0.5 LPM with goal to achieve 2 LPM Consider increasing anticoagulation therapy to facilitate low flows. Do not flow below 0.5 LPM Once flows of 2 LPM, wean FiO_2_ to 0.5. Consider off ECMO trial at this point Goal is to achieve PaO_2_ > 60 mm Hg with vent FiO_2_ < 0.5 and Insp Plat pressure < 30 cm H_2_O for 12–24 h If successful, then consider decannulation	Process described only for VA-ECMO	Turn down sweep and FiO_2_ increments of 0.5 LPM and FiO_2_ 0.1 while following ABG to maintain PaO_2_ > 60 and PaCO_2_ to target pH 7.35–7.45 Successful trial of off ECMO when FiO_2_ and sweep can be maintained at 0 for > 30 min	Process described only for VV-ECMO

ABG, arterial blood gas; ECMO, extracorporeal membrane oxygenation; FiO2, fraction of inspired oxygen; Insp Plat, inspiratory plateau pressure; LPM, liters per minute; PaCO_2_, partial pressure of carbon dioxide; PaO_2_, partial pressure of oxygen; PEEP, positive end-expiratory pressure; Pplat, plateau pressure; SaO2, oxygen saturation, VA, veno-arterial; VV, veno-venous.

### Referral process and patient selection

Program documents contained similar inclusion/exclusion criteria across programs (Table 3, Table 4, Table 5). Specific inclusion/exclusion criteria of each institution are listed in Tables 4 and 5. The referral process for ECMO is initiated through cardiac surgery at most institutions. A limited number of institutions have protocols in place for automatically notifying all team members once a potential candidate for ECMO has been identified.

### Anticoagulation and ventilator management

Most programs used unfractionated heparin for routine anticoagulation (Tables 6 and 7). One program used low-molecular-weight heparin as a subcutaneous injection for daily maintenance in select patients. Variation was seen in bolus dosing for cannulation (5000-30000 units), monitoring (activated clotting time vs partial thromboplastin time [PTT]) and targets for maintenance (activated clotting time: 160-250 s; partial thromboplastin time: 50-70.9 s). Only one program specifically addressed monitoring and management of coagulopathy.

Ventilator strategies generally focused on initial parameters rather than guidance related to a more longitudinal strategy (ie, maintenance and weaning). Most programs suggested an initial “lung protective strategy”; however, variation occurred in how this was defined (peak inspiratory pressure: 20-30 cm H_2_O; positive end expiratory pressure: 5-15 cm H_2_O; tidal volume: 3-4 vs 4-6 mL/kg; inspired fraction of oxygen 0.3-0.5). Some programs specified only that management should be left to the discretion of the treating team. No programs specifically addressed ventilation strategy for VA-ECMO.

### Weaning protocol

Most programs described a strategy to safely facilitate a weaning trial and recommended daily assessment for consideration of weaning (Tables 8 and 9). Programs provided more-specific criteria for determination of successful weaning from VV-ECMO compared to VA-ECMO. For VA-ECMO, programs suggested echocardiographic assessment on very low flow (1.5 LPM). One program (institution 4) provided specific hemodynamic and echocardiographic criteria for consideration of a successful VA-ECMO wean.

## Discussion

Using a Delphi process involving key stakeholders from across Canada, 37 key elements spanning 5 domains that should be included in ECLS protocols were identified. These key elements represent important areas to be addressed by an institution delivering ECLS services. The prevalences of key elements in existing ECLS protocols from 13 Canadian institutions were then assessed. Only 5 key elements were present in more than 50% of protocols. The prevalence of key elements in documentation varied across centres, with a higher prevalence found in centres with heart and/or lung transplantation programs. Given that the majority of ECLS cases are done at lower-volume centres in Canada,^4^ and lower-volume centres have been shown to have worse outcomes,^5^ this study identified potential areas for quality-improvement initiatives aimed at increasing protocol completeness and harmonization across institutions.

The identification of domains and key elements of ECLS provides a workable framework for program development and quality-improvement initiatives. Although programs may use the key-element framework to develop local protocols, this study could be taken as an opportunity to begin collaborating on a national ECLS program manual and outcome sharing. This collaboration should promote information sharing across programs and reduce the burden on individual institutions as program development evolves.

Many important areas of the ECLS process remained unaddressed by the majority of institutions. Key elements in the initiation and program administrative domains were underrepresented. Program administration, including a defined process for program review, quality improvement, and team education are essential tenets of the ELSO Centre of Excellence Criteria.^17^ Key elements dealing with urgent and emergent aspects of ECLS delivery (ie, initiation, emergency troubleshooting, etc.) should be protocolized. Predictable emergencies should not be dealt with on an ad hoc basis. A common practice in other industries is to develop protocols for predictable high-stakes events.^8^^,^^9^ Although key elements in these areas were underrepresented, some programs did have robust protocols that could be adapted to other institutions. National collaboration on program development using the key-element framework would allow centres to share best practices and learn from other institutions.

We found that heart and/or lung transplantation centres had more complete protocols. Given that transplantation centres also have a higher volume of ECLS cases, compared with nontransplantation centres, protocol completeness likely reflects the need for a clearly defined structure and organization with respect to roles and responsibilities of ECLS stakeholders at each centre. Lower-volume centres may rely on a more ad hoc approach to delivery of ECLS services when cases arise. Although direct evidence linking protocol completeness and better ECLS outcomes at higher volume centres is lacking, national collaboration and sharing of best practices among institutions may be one avenue to ensure that similar high-quality care is delivered at all Canadian institutions.

The narrative review of the 5 common key elements provides readers with a summary of how institutions approached these components of ECLS delivery. Key elements with significant consensus may indicate higher-quality evidence or agreement on an accepted standard of care. For example, agreement was reached on the criteria for patient selection, which may reflect accepted transplantation criteria.^18^ However, key elements with variability may represent areas of uncertainty. This uncertainty is evident in the key elements addressing anticoagulation and ventilation. Anticoagulation strategies show variation in drugs, dosing, and monitoring. Few programs addressed the management of coagulopathy associated with ECLS. This variability is seen internationally and may reflect uncertainty in the evidence related to anticoagulation on ECMO.^19^, ^20^, ^21^ Similar uncertainty remains around ideal ventilatory practices for VV- and VA-ECMO.^12^^,^^22^^,^^23^ This variation in practice may represent an opportunity for future research and quality-improvement initiatives. It also highlights the need for outcome tracking in order to effectively implement such a program.

This study faced several limitations. The Delphi panel represented many of the institutions that shared documentation for the study, and the representative sample chosen may have led to sampling bias. This bias was minimized by ensuring representation from all provinces and also that at least 2 samples from each type of institution (lung and heart transplantation capable, heart transplantation capable, and cardiac surgery-only capable) were represented. Additionally, better characterization of the participating centres (eg, ECLS volume, academic vs community, geographic location) could have enriched the analysis and represents a limitation of this study. Also, it was impossible to verify that all program documentation was shared. Using ECLS leads or physicians with significant involvement in ECLS programs who would have the most familiarity with documentation should have mitigated this shortcoming. Finally, clinical practice may vary from what is described in program documentation.

## Conclusion

Using an interdisciplinary panel, 37 key elements across 5 domains were identified that should be incorporated into ECLS protocols. Assessment of program documentation from Canadian institutions showed variability in the number of key elements included in protocols and how protocols addressed specific key elements. Further exploration of this variability could improve clinical care. The key-element framework provides an opportunity for national collaboration on ECLS program development.
